# The redistributive effect of the public health system: the case of Sierra Leone

**DOI:** 10.1093/heapol/czad100

**Published:** 2023-11-07

**Authors:** Jacopo Gabani, Sumit Mazumdar, Sylvester Bob Hadji, Michael Matthew Amara

**Affiliations:** Centre for Health Economics, University of York, Alcuin Block A, Heslington, York YO10 5DD, UK; Department of Economics and Related Studies, University of York, Heslington, York, YO10 5DD, UK; Centre for Health Economics, University of York, Alcuin Block A, Heslington, York YO10 5DD, UK; Department of Economics, University of Sierra Leone, Fourah Bay College, Mount Aureol, Freetown, Sierra Leone; Ministry of Health and Sanitation, Government of Sierra Leone, 4th & 5th Floor, Youyi Building, Brookfields, Freetown, Sierra Leone

**Keywords:** Health systems, equity, health financing, redistribution, fiscal incidence

## Abstract

Universal health coverage (UHC), health equity and reduction of income inequalities are key objectives for the Sierra Leone government. While investing in health systems may drive economic growth, it is less clear whether investing in health systems reduces income inequality. Therefore, a crucial issue is to what extent the Sierra Leone public healthcare system reduces income inequality, and finances and provides healthcare services equitably. We use data from the Sierra Leone Integrated Household Survey 2018 to complete a financing and benefit incidence analysis of the Sierra Leone public healthcare system. We extend these analyses by assessing the redistributive effect of the public healthcare system (i.e. fiscal incidence analysis). We compute the redistributive effect as the change in Gini index induced by the payments for, and provision of, public healthcare services. The financing incidence of the Sierra Leone public healthcare system is marginally progressive (i.e. Kakwani index: 0.011*, *P*-value <0.1). With regard to public healthcare benefits, while primary healthcare (PHC) benefits are pro-poor, secondary/tertiary benefits are pro-rich. The result is that overall public healthcare benefits are equally distributed (concentration index (CI): 0.008, not statistically different from zero). However, needs are concentrated among the poor, so benefits are pro-rich when needs are considered. We find that the public healthcare system redistributes resources from better-off quintiles to worse-off quintiles (Gini coefficient reduction induced by public healthcare system = 0.5%). PHC receives less financing than secondary/tertiary care but delivers a larger reduction in income inequality. The Sierra Leone public healthcare system redistributes resources and reduces income inequality. However, the redistributive effect occurs largely thanks to PHC services being markedly pro-poor, and the Sierra Leone health system could be more equitable. Policy-makers interested in improving Sierra Leone public health system equity and reducing income inequalities should prioritize PHC investments.

Key messages

- Investments in public healthcare systems are widely seen as improving countries’ economic growth prospects. However, it is less clear to what extent they also improve income inequalities by redistributing resources from rich to poor.
- Benefit and financing incidence analysis can provide this information when they are done together. We complete a benefit, financing and fiscal incidence analysis with data from the Sierra Leone Household Integrated Survey 2018.
- We find that the public healthcare system could be more equitable, and that it redistributes resources from rich to poor improving inequality.
- The primary healthcare (PHC) system level is the main driver of this redistribution: policy-makers interested in improving Sierra Leone public health system equity and redistributive effects should prioritize PHC investments.

## Introduction

Numerous countries have embarked on health system reforms to accelerate progress towards universal health coverage (UHC) (Cotlear *et al.*, 2015; Cotlear and Rosemberg, 2018), the aspiration that their entire populations can access the services they need equitably, without incurring financial hardship (World Health Organization, 2019). Sierra Leone has explicitly stated UHC and equity as goals in the recently approved Ministry of Health and Sanitation (MoHS) National Health Sector Strategic Plan 2021–2025, and improved primary healthcare (PHC) is a key strategy to reach those objectives (Sierra Leone Ministry of Health and Sanitation, 2021; Brundtland, 2022). The MoHS considers PHC financing as a critical priority in reaching these goals, recognizing its role as a cornerstone of UHC (Binagwaho and Ghebreyesus, 2019). Moreover, reducing income inequalities is also an explicit target of the Sierra Leone Medium Term National Development Plan 2019–2023 (Government of Sierra Leone, 2019).

Although there is some evidence that public health expenditure support economic growth (Remes  *et al.*, 2020; Wang, 2015; Piabuo and Tieguhong, 2017; Raghupathi and Raghupathi, 2020; Gaies, 2022), the impact of public health expenditure on inclusive growth and income inequality is less understood. Policymakers have limited knowledge regarding the extent to which the Sierra Leone public healthcare system is financed progressively or regressively, provides healthcare benefits to the population according to their needs and redistributes resources among different socio-economic groups.

Therefore, the main research question of this paper is whether the Sierra Leone public healthcare system redistributes resources and is equitable in health financing and benefits provision. To answer this question, we adopt the definition of an equitable system provided by Ataguba and Akazili (Ataguba and Akazili, 2010), which encompasses progressive health financing and benefits provision based on needs. We run financing (Ataguba *et al.*, 2018), benefit (McIntyre and Ataguba, 2011) and fiscal incidence (Lustig, 2015; 2019) analyses focused on the public healthcare system.

It is important to run financing and benefit incidence analysis together (Huang *et al.*, 2007; Ataguba and McIntyre, 2012; Mills *et al.*, 2012) because, according to the chosen definition of equitable health system (Ataguba and Akazili, 2010), assessing the equity of the Sierra Leone public health system requires an understanding of who bears the health financing burden and who receives healthcare benefits. For example, if financing for the public healthcare system is progressive (or regressive), but the distribution of benefits is pro-rich (or pro-poor), then we cannot conclude that the public healthcare system is equitable. These insights can also inform political economy implications of health policies aimed at improving equity.

We also examine the redistributive effect of the public healthcare system, defined as the change in income inequality induced by the public healthcare system (Lustig, 2015; Higgins and Lustig, 2016; Abad and Lindert, 2017; Inchauste and Lustig, 2017a). We measure the Gini index before and after public health financing and public healthcare benefits are considered to understand whether the public healthcare system reduces the Gini index of income inequality. The change in Gini index induced by the public health sector is an indicator included in the Sustainable Development Goals (indicator 10.4.2, which refers to the redistributive effect of all government sectors, not only the health sector).

This paper primarily focuses on the public healthcare system, including health financing and provision of benefits, so that our findings are more actionable for policy-makers. Given the regressivity of private out-of-pocket (OOP) expenditures (Asante *et al.*, 2016) and their importance in financing the Sierra Leone health sector, we extend the analysis including private OOP expenditures and private sector healthcare providers in robustness checks.

### The Sierra Leone health system

Before presenting our methods, we provide a brief introduction of the Sierra Leone health system. Sierra Leone is administratively organized in regions, which are divided in districts, and its health system is organized in three levels. The PHC system level is served by peripheral health units (PHUs), encompassing maternal and child health posts (the health facility that is closest to the community) and larger health centres, which can provide basic emergency obstetric and neonatal care, among other services (Huang *et al.*, 2007). The secondary level includes regional level and district level hospitals. Finally, the tertiary level includes Connaught Hospital, Ola During Children Hospital and Princess Christian Maternity Hospital (Koka *et al.*, 2016). This information is important to understand how to use the unit costs provided by National Health Accounts (NHA) 2018 to measure the cost of services utilized by households, as recorded in Sierra Leone Integrated Household Survey (SLIHS) 2018 (further details on this below).

Health expenditure in Sierra Leone is largely financed by households’ OOP health expenditures (55% of total health expenditure [THE]), followed by external health expenditure (30% of THE) and government public health expenditure (14% of THE) (World Bank, 2019). The remaining 1% is pre-paid private domestic health expenditures. Government expenditure is largely financed by taxes, excises, duties and other domestic revenues, and from external on-budget financing. In the ten years before 2018 (i.e. 2007–2017 period), OOP as % THE decreased and government expenditure as % of THE increased, a pattern similar to the so-called ‘health financing transition’ (Fan and Savedoff, 2014; Gabani *et al.*, 2023). As the sources of external resources are taxpayers of countries providing development assistance for health, these resources have been ignored in our analysis (Ataguba *et al.*, 2018). In terms of expenditure allocation, government health expenditure in 2018 primarily focused on human resources (54% of the government health budget), followed by goods and services, including drugs (35%), and transfers to the PHC level (7%) (Government of Sierra Leone, 2020). According to the System of Health Accounts 2011 framework, which includes the healthcare provider (HP) module, the NHA 2018 reveals that hospital expenditures constituted the largest share (39% of THE), followed by ambulatory and preventive care providers (33%) and health system governance, financing and administration costs (24%). Using the System of Health Accounts 2011 framework, which includes the healthcare provider (HP) module, the NHA 2018 reveals that hospital expenditures constituted the largest share (39% of THE), followed by ambulatory and preventive care providers (33%) and health system governance, financing and administration costs (24%). A more detailed table is provided in Appendix 1.

## Methods

### The data

We use households’ total expenditure per adult equivalent as the living standards measure to rank and group households in five socio-economic groups: from the lowest income quintile (#1) to the highest (#5). Official adult equivalences for Sierra Leone are provided by the SLIHS 2018. All analyses use survey household and population weights as relevant and as provided by the SLIHS 2018. Data related to direct and indirect taxes are from the SLIHS 2018, and all tax-related assumptions are based on documents from the Sierra Leone National Revenue Authority. From now on, and although it is recognized that hospitals might provide PHC services, we follow the Sierra Leone definition (Government of Sierra Leone, 2015) that the PHC health system level is approximated to be the PHU health system level.

The data source for utilization (i.e. number of visits made by households, at secondary/tertiary hospitals and at PHUs) is the Sierra Leone Integrated Household Survey (SLIHS) of 2018 (Statistics Sierra Leone, 2018), a living standards survey. For costs of services the main source is the Sierra Leone National Health Accounts (NHA) 2018, which had estimated costs for outpatient and inpatient services delivered at different levels of the health system (health centres/primary level, secondary and tertiary level hospitals) (WHO, 2021). Finally, the official ‘Government of Sierra Leone Budget for Fiscal Year 2020’ from the Sierra Leone Ministry of Finance detailing actual revenues collected, health sector budget allocations and public health expenditures, for the year 2018, was used for adjusting the total value of benefits and financing for health, as detailed in the next sections.

To estimate the redistributive effects of the public healthcare system, we first conduct two primary underpinning analyses: (1) financing incidence analysis and (2) benefit incidence analysis.

### Financing incidence analysis

We estimate direct income taxes, goods and services tax and fuel excises and duties, paid by each household, using SLIHS 2018. We group goods and services taxes and fuel excises and duties under ‘indirect taxes’. Each household direct and indirect tax contribution has been computed using the assumptions in Table 1, and additional details are provided in Appendix 1.

**Table 1.** Assumptions and computations for tax used as public health financing sources

Tax	Assumptions and computation
Direct income tax (26% of total domestic government revenues)	First, we measure income earned by all members of households who declared having a formal employment contract. Second, we apply to that income the rates stated by the Sierra Leone National Revenue Authority in the Tax and non-tax revenues guide 2019, to derive income tax revenue.
Goods and services tax (GST) (20% of total domestic government revenues)	We use, for all reported purchased goods, annualized, the standard National revenue Authority rate of 15%. The only exceptions made are local rice and imported rice, as well as other items as per Sierra Leone Revenue authority rules (e.g. printed materials, insurance services), which are GST exempt and for which we compute zero GST.
Excise and duties on petroleum products (8% of total domestic government revenues)	We assume that fuel tax charged on retail gasoline purchased by households is 9% of the retail value, as reported in a World Bank/Statistics Sierra Leone 2014 report (179). In addition, we assume that 30% of the ticket paid by households when they use taxi, minibuses, motorbikes and any other transport is fuel.

Source: authors’ elaboration.

The tax revenue estimates from SLIHS have been compared with the Sierra Leone official Ministry of Finance revenues: in case of discrepancies, the difference was allocated across households following their proportional contribution to each tax estimated via the SLIHS data (Ataguba *et al.*, 2018) (e.g. our estimate for all indirect taxes was 13% below the official Ministry of Finance figure, so the indirect tax estimated was increased by that amount, and the increase distributed proportionally to households following the distribution measured via SLIHS 2018). Indirect and direct tax are 79% of total domestic revenues: we note that all other government revenues (e.g. corporate income tax and mines department revenues) were assumed to have the same households’ distribution measured for direct and indirect tax via SLIHS (O’Donnell *et al.*, 2007). More details on all assumptions made are shown in Appendix 1. To assess progressivity of public health financing, we present comparisons of contributions to the public healthcare system across income quintiles, concentration curves and indexes and Kakwani indexes (Kakwani, 1977; O’Donnell *et al.*, 2007; Ataguba *et al.*, 2018).

For the financing incidence analysis, the concentration curves show the cumulative share of taxes contributed by households ranked by our chosen living standard measure (i.e. total household expenditure per adult equivalent). CIs are computed as twice the area between the concentration curve and the line of equality (i.e. a straight 45° line), which represents the concept of health taxes being exactly equally distributed across different living standards. Formally, the CI (O’Donnell *et al.*, 2007):


(1) $$CI(T\,|\,Y) = \frac{2}{{\bar T}}cov\left( {{t_i},{R_i}} \right)$$

where $T$ represents contributions to financing health by household $i$, $Y$ represents the living standards measure of household expenditure per adult equivalent, $R$ the fractional rank of household $i$ (which by definition has mean 0.5), ranked by the living standards measure $Y$ (expenditure per adult equivalent). The index is negative if taxes are regressive (concentrated among poorer households) and positive if taxes are progressive (concentrated among richer households). The index was calculated using the conindex Stata command (O’Donnell *et al.*, 2016).

Finally, the Kakwani index (Kakwani, 1977) is twice the area between the taxes (or any other) concentration curve, and the living standards concentration curve (i.e. the Lorenz curve). For this reason, when showing health financing concentration curves, we will also show the Lorenz curve. The Kakwani index can be computed as the difference between the CI of interest, in our case total contributions to health, and the Gini index. Finally, it can be computed as the coefficient $\beta $ in the following convenient regression (O’Donnell *et al.*, 2007):


(2) $$2\sigma _R^2\left[ {\frac{{{t_i}}}{{\bar T}} - \frac{{{y_i}}}{{\bar Y}}} \right] = \alpha + \beta {r_i} + {u_i}$$

where ${t_i}$ is contributions to financing health made by household $i$, ${y_i}$ is the living standards measure of household expenditure per adult equivalent for household $i$, ${r_i}$ is the fractional rank of household $i$ in the household expenditure per adult equivalent distribution. This regression method allows us to estimate the Kakwani index standard error (SE) as well, and it is the method used in this paper to compute Kakwani indexes. The Kakwani index for all health financing contributions is measured as the weighted average of the Kakwani indexes (Ataguba *et al.*, 2018) of each tax source, with weights (see Appendix 1) informed by the official budget documents of the Government of Sierra Leone (Government of Sierra Leone, 2020; 2021).

Although the focus of the analysis is the public healthcare system, we extend the financing incidence analysis by including households OOP health expenditures (see Appendix 2). When extending the analysis to include OOP health expenditures, we use NHA 2018 weights for government health expenditures (9% of THE) and OOP health expenditures (56% of THE) to measure the Kakwani index.

### Benefit incidence analysis

In order to measure benefit incidence, we implement the following steps (McIntyre and Ataguba, 2011):

- Estimate households’ benefit utilization. SLIHS provides detail of outpatient and inpatient visits at public hospitals, and at PHUs, which are health facilities responsible for PHC service delivery. Many households reported inpatient services at PHUs: it is possible that patients remained overnight at the largest PHUs (community health centres [CHCs]). Outpatient services recall period was 4 weeks, and so the households’ utilization was annualized: all outpatient visits were multiplied by 13 to represent a period of one entire year (i.e. 52 weeks). For inpatient services there was no annualization as the recall period was one year.
- Using government THE for inpatients and outpatients’ services at hospitals and PHUs, from NHA 2018, we compute the unit cost per service. This is measured as THE for inpatients services divided by ‘quantity of public healthcare inpatients benefits (nights) utilized’ from SLIHS 2018, or THE for outpatients’ services divided by ‘quantity of public healthcare outpatients’ benefits (episodes) utilized’, from SLIHS 2018. To compute the government share of THE for inpatient and outpatient services by hospitals and PHUs, we used the government share of hospital and PHU health expenditure. Unit costs computed in this way are provided in the next section, and more details are provided in Appendix 1.
- We compute the US$ value of the benefits received by each household as the multiplication of ‘quantity of public healthcare benefits utilized’ from SLIHS 2018 and ‘public healthcare benefit unit cost’ from the previous step. The benefit received by a given income quintile group is the sum of the benefits received by all households in that income quintile group.
- Finally, we compute the public subsidy by subtracting direct user fees paid by each household to the provider to access the services (i.e. consultation fees—which may be informal and used to finance volunteer healthcare workers [Witter *et al.*, 2016]). As common in other benefit incidence analyses (Wagstaff, 2012), we truncated the public subsidy to zero when subtracting OOP spending resulted in a negative public subsidy. Henceforth, we will refer to public subsidy and public benefits interchangeably^1^.

Formally, we measure public subsidies $b$ per households $i$ as follows (O’Donnell *et al.*, 2007):


(3) $${b_i} = \mathop \sum \limits_k {\alpha _k}\left( {{q_{ki}}{c_k} - {f_{ki}}} \right)$$

where ${q_{ki}}$ is the quantity of service $k$ utilized by household $i$, ${c_k}{\mathrm{\,}}$is the unit cost of service $k$, ${f_{ki}}$ are direct user fees paid by household $i$ to access service $k$ and ${\alpha _k}$ is an annualization factor, equal to 1 for inpatient services (recall period in SLIHS 2018: one year) and 13 for outpatient services (recall period in SLIHS 2018: 4 weeks).

Because there is no health expenditure for inpatient services at PHUs, but households reported inpatient services at PHUs, the unit cost for inpatient services at PHUs has been assumed to be the average between PHUs outpatient unit costs and hospitals inpatient unit costs.

Benefits values by household are then used to assess pro-richness or pro-poorness of public healthcare benefits (i.e. subsidies) distribution. We compare the total value of benefits received by each income quintile group. As a robustness check, we use WHO CHOICE 2021 data to compute the unit costs and total value of benefits (Appendix 2). Concentration curves and indexes^2^ are produced for total benefits, outpatient PHU and hospital services and inpatient PHU and hospital services, for a total of five curves. We note that standard CIs provide a measure of relative inequality (Erreygers and van Ourti, 2011). For this reason, and in addition to graphs of benefits across quintiles, generalized CIs are provided in Appendix 2.

To complete the equity analysis, healthcare needs have to be considered. In absence of a subjective health well-being measure in SLIHS 2018, we compute healthcare need by household in the following way (McIntyre and Ataguba, 2011): for each household member, the variable ‘health need’ is valued as one (=1) if the household member reported being sick or injured in the past 4 weeks, or if the household member had to consult a healthcare provider for reasons other than being sick or injured. This definition of healthcare need assumes that healthcare need is equal across individuals, regardless of income, age, gender or health conditions. Healthcare need at the household level is computed as the sum of the healthcare need variable for all household members.

First, we compare the distribution of needs across quintiles, and we compare this distribution to the distribution of all public healthcare benefits. Second, we provide a concentration curve for healthcare needs. Finally, we measure the ‘benefits need index’ (also referred to as horizontal inequity [Wagstaff and van Doorslaer, 2000]), which is the difference between the CI of benefits (all levels considered separately) and the CI of need (Wagstaff and van Doorslaer, 2000).


(4) $$C{I_{BN}} = C{I_{Benefits}} - C{I_{Need}}$$

Because CIs can go from −1 to +1, $C{I_{BN}}$ can range from −2 and +2^3^, and represents the extent to which public healthcare benefits provision is proportional, pro-poor or pro-rich when compared with healthcare need.

As CIs can be measured as regressions coefficients via the convenient regression (O’Donnell *et al.*, 2007), we test the hypothesis that the difference between two CIs is zero via the following formula (Clogg *et al.*, 1995):


(5) $$Z = \frac{{C{I_1} - C{I_2}}}{{\sqrt {SE{{(C{I_1})}^2} + SE{{\left( {C{I_2}} \right)}^2}} }}$$

Finally, we identify the determinants of the CI of public healthcare benefits using recentred influence functions (RIF) (Firpo *et al.*, 2009; Heckley *et al.*, 2016; Rios-Avila, 2020). Intuitively, each household has a RIF value which represents the household’s influence on the CI. Given this premise, the mean of the RIF is equivalent to the CI. This allows for ordinary least squares (OLS) mean regression analyses: RIF values form the dependent variable, and covariates coefficients can be interpreted as the covariates’ effect on the CI of a marginal increase in the mean of the covariate, if the covariate is continuous, or an increase in proportion of individuals in a certain group, if the covariate is a dummy. For a binary variable (e.g. household residing in rural equal one, zero otherwise), the CI percentage contribution (i.e. marginal effect) of an increase of 1 percentage point in the proportion of households belonging to a particular group (e.g. household residing in a rural area) is calculated as $\frac{\beta }{{CI}}*1\% $, where $\beta $ is the binary variable OLS coefficient.

**Figure 1.**
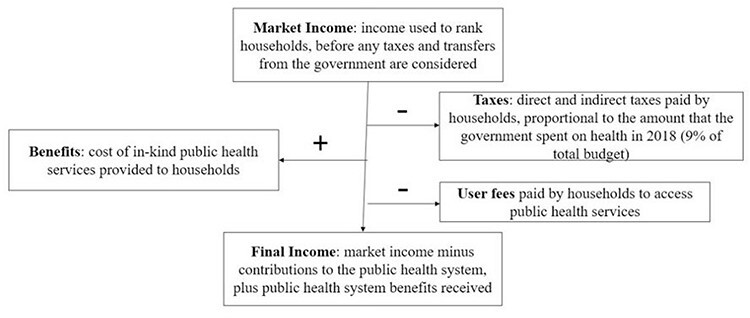
From market income to final income

Source: authors, revising and simplifying from Lustig (2015) and Inchauste and Lustig (2017a).

Two steps are required for this analysis. First, the computation of CI RIF values for each household. Second, covariates of interest (i.e. age of the head of household [HHH], rural/urban residence, education of the HHH, income quintile, employment situation of the HHH and gender of the HHH) are regressed on CI RIF values. SEs are bootstrapped as suggested in the relevant RIF-CI-OLS literature (Firpo *et al.*, 2009; Rios-Avila, 2020). We present both unweighted and weighted OLS results, in line with the relevant literature on regression weighting (Solon *et al.*, 2015). We describe in more detail the procedure and its benefits versus other decomposition methods (Wagstaff *et al.*, 2003) in Appendix 3.

While the focus of the analysis is the public healthcare system, we extend the benefit incidence analysis by including private healthcare providers (see Appendix 2).

### Measuring the redistributive effect of the public healthcare system

We assess the redistributive effect of the public healthcare system in three steps. First, we compute ‘net benefits’ (Lustig, 2015; Inchauste and Lustig, 2017a) for each household as the difference between the public healthcare subsidy received by the household and the estimated contribution made by the household to public healthcare financing (Figure 1). Net benefits across socio-economic groups and the percentage of resources redistributed over total benefits/financing show whether the public healthcare system is re-distributing resources between better-off and worse-off households.

Second, we measure the Gini index of income inequality before and after public health financing (see eq. (6)), as in O’Donnell *et al.* 2007, Box 17.1 (O’Donnell *et al.*, 2007): the change in Gini index measured via eq. (6) represents the redistributive effect of public healthcare financing.


(6) $$\begin{aligned}R{E_{Public\ health\ financing}} = \,& {G_{Market\ income}} \nonumber\\ & - {G_{Market\ income - health\ financing}}\end{aligned}$$

Third, we measure the Gini index of income inequality before and after health financing ‘and’ public subsidies, as in Lustig 2015 (Lustig, 2015): the change in Gini measured via eq. (7) represents the change in income inequality driven by the public healthcare system (‘marginal contribution’ in [Lustig, 2015; Inchauste and Lustig, 2017b]).


(7) $$\begin{aligned}& R{E_{Public\ health\ system}} \nonumber\\ & = {G_{Market\ income}} \nonumber\\ & \quad\ -\ {G_{Final\ income = market\ income - health\ financing\ +\ public\ subsidies}}\end{aligned}$$

where $G$ stands for Gini index, market income is income before any health financing contributions are collected or health subsidies are provided and final income is household expenditure by adult equivalent minus public health financing contributions plus public healthcare subsidies. Via eq. (7), we compute the redistributive effect of the entire public health system, the redistributive effect of the public PHC system and the redistributive effect of public secondary/tertiary healthcare system.

If the public healthcare system is redistributing resources from richer to poorer households, then the final income of poorer households will be larger than their market income.

We refer the reader to the numerous online sources explaining how to measure the Gini index, and we measure it using the ‘conindex’ Stata command (O’Donnell *et al.*, 2016). Stata 17, survey weights and adult equivalence factors have been used for all analyses. SEs are robust and clustered.

**Figure 2.**
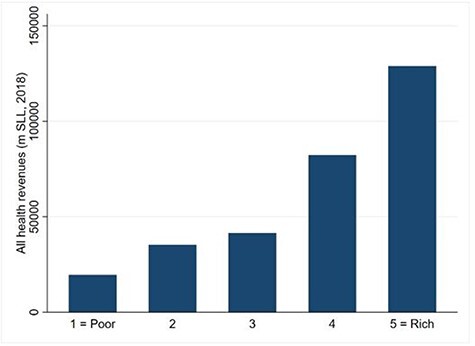
Public financing incidence analysis

Source: authors’ elaboration.

## Results

### Financing incidence analysis

The Sierra Leone public healthcare system is mostly financed by contributions from the richest quintile (Figure 2), as the richest quintile pays for highest share of the public health financing contributions when compared with other socio-economic groups.

**Figure 3.**
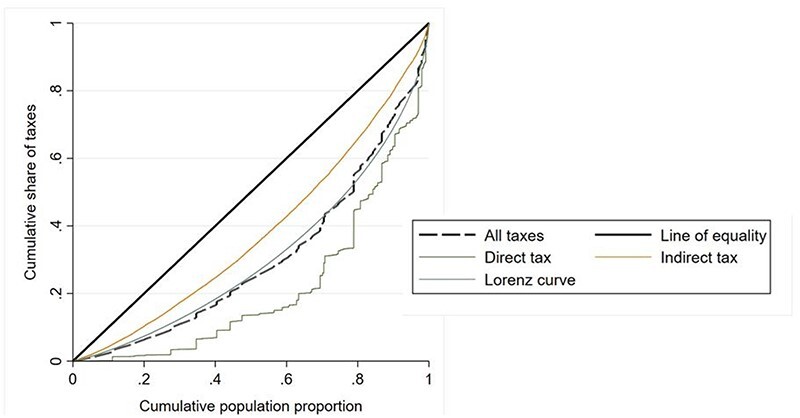
Concentration curves for direct and indirect tax revenues

Source: author calculation, following the concentration curve definition provided in the methods section.

The CIs and curves show that all of the analysed financing sources are concentrated among the richest quintiles, and that this concentration is stronger for direct taxes rather than for indirect taxes. Figure 3 also shows that the concentration curve for total public health financing contributions (‘all taxes’) and the Lorenz curve cross each other multiple times.

The Kakwani index of health financing contributions across all taxes (Table 2, 0.011*, *P*-value <0.1) show that public health financing contributions are collected in a way that is only very moderately progressive. The Kakwani indexes by tax sources (Table 2) suggest that this overall result is driven by progressivity of direct taxes and regressivity of indirect taxes.

**Table 2.** Concentration and Kakwani indexes for sources of public financing for health

	Concentration index	Kakwani index
Total financing	0.393^***^	0.011^*^
Direct tax	0.569^***^	0.188^***^
Indirect tax	0.242^***^	−0.139^***^

Source: authors’ calculation. Robust SEs have been used.

*P* < 0.1*, *P* < 0.5** and *P* < 0.01***. For completeness, the Gini index is 0.381***.

In Appendix 2 we extend the financing incidence analysis including OOP health expenditures. The Kakwani index is the weighted average (Ataguba *et al.*, 2018) of the Kakwani indexes for the public healthcare system and OOP health expenditures from NHA 2018. When OOP health expenditures are included, the overall health financing in Sierra Leone becomes regressive due to the regressivity of OOP health expenditures.

Table 2. Concentration and Kakwani indexes for sources of public financing for health

### Healthcare benefits incidence analysis

We start by presenting computed services values from NHA 2018 and SLIHS 2018 in Table 3.

**Table 3.** Unit costs by service and definition/computation in NHA 2018 and SLIHS 2018

NHA health expenditure definition	SLIHS 2018 definition	Computed value (US$) from NHA 2018
Ambulatory care provider, outpatient care	PHUs outpatient	0.34
Not available	PHUs inpatient	2.39
Hospitals, outpatient care	Hospitals outpatient	1.89
Hospitals, inpatient care	Hospitals inpatient	4.45

Source: authors’ elaboration. Values from: NHA, 2018, as described in the methods section.

**Figure 4.**
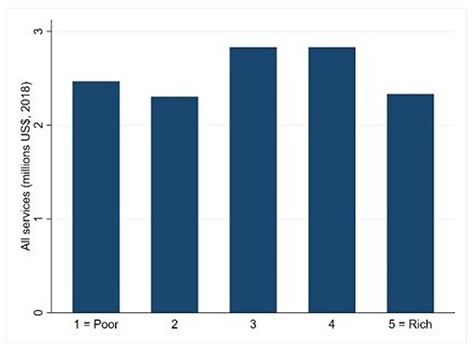
Benefit incidence across income quintiles, for all services (PHUs and hospitals, inpatient and outpatient)

Source: authors’ elaboration.

The distribution of public healthcare benefits (i.e. subsidies) across quintiles is presented in Figure 4. Healthcare benefits were rather equally distributed in 2018, and there is no evident pro-rich or pro-poor bias. In other words, it appears that a similar amount (in value) of public services is delivered across the five income quintiles, except slightly lower benefits for the richest quintile.

**Figure 5.**
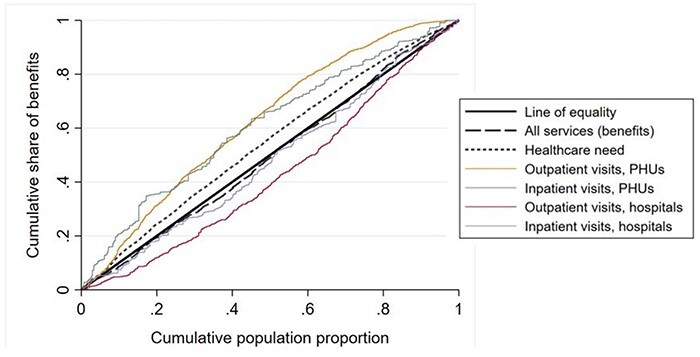
Concentration curves for healthcare needs, total benefits, PHU inpatient benefits, PHU outpatient benefits, hospital inpatient benefits and hospital outpatient benefits

Source: authors’ calculation, following the concentration curve definition provided in the methods section.

The distribution of public benefits for outpatient and inpatient hospital and PHU services is represented by the CIs in Table 4 and relative curves in Figure 5. The results confirm that the overall public healthcare benefits are distributed equally (CI: 0.008).

**Table 4.** Concentration indexes for public healthcare benefits

Public benefits	Concentration index (CI)
All public benefits	0.008
Inpatient hospital	0.037
Outpatient hospital	0.143^***^
Inpatient PHU	−0.220^***^
Outpatient PHU	−0.247^***^

Source: authors’ calculation. Robust SEs have been used; *P* < 0.1*, *P* < 0.5** and *P* < 0.01***.

The small pro-poor bias of total services is a result of two different patterns: while PHU services are pro-poor (outpatient and inpatient PHU benefits CI: −0.248, *P* < 0.01 and −0.220, *P* < 0.01, respectively), hospital outpatient services are pro-rich (outpatient hospital benefits CI: +0.143, *P* < 0.01), and hospital inpatient services show a non-significant and limited pro-rich bias (inpatient hospital benefits CI: +0.037).

To ensure robustness of our results, we conduct additional checks (see Appendix 2). We consider additional OOP costs that patients paid to providers, such as drugs and tests. These costs are unlikely to have been remitted to the central level, are not rent extracted by providers and therefore were not considered in the main analysis given that the objective is to measure public subsidies (O’Donnell *et al.*, 2007). However, it might be argued that they should be considered. The resulting CIs are consistent with the main analysis results shown in Table 4. In a second robustness check, we use unit costs from WHO CHOICE 2021 instead of unit costs computed from NHA 2018. The results are again largely similar to our main results. However, the distribution of overall benefits is slightly pro-poor rather than being equally distributed. This is driven by the difference in unit costs: hospital services in WHO CHOICE 2021 are less expensive when compared with PHU services. NHA unit costs are collected from government, development partners and household surveys, and therefore are to be preferred because WHO CHOICE unit costs are modelled unit costs, rather than actually collected.

In addition to analysing benefits by income quintile, we explored the distribution of benefits across the 16 districts of Sierra Leone (see Appendix 2). While public benefits varied across districts, there was no notable concentration of benefits in the most urban district, which encompasses tertiary hospitals and the capital city (Western Area Urban). This reinforces the finding that public benefits are not significantly pro-rich or pro-poor. Notably, the districts of Falaba and Pujehun exhibited the lowest public benefits per capita. The limited public benefits in Falaba may be attributed to the absence of a district hospital, whereas the situation in Pujehun may be due to its low population density and high percentage of rural population, potentially restricting access to hospital services (UN Office for the Coordination of Humanitarian Affairs, 2015).

**Figure 6.**
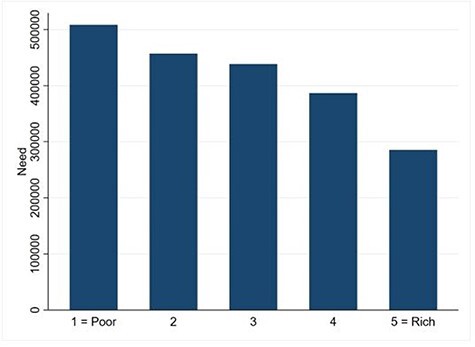
Healthcare need across quintile groups

Source: authors’ calculation.

Figure 6 shows that needs are concentrated among poorer households. The CI of health needs (Table 5, −0.091, *P* < 0.01) confirms this finding. However, we note that self-reported healthcare need is likely underestimating the actual need of poorer households (Sauerborn *et al.*, 1996; Mcintyre *et al.*, 1998; McIntyre and Ataguba, 2011).

**Table 5.** Concentration indexes and benefits needs index

Public benefits	Benefits (CI)	Needs (CI)	Benefits needs index
All public benefits	0.008	−0.091^***^	0.099^***^
Inpatient hospital	0.037	−0.091^***^	0.128^**^
Outpatient hospital	0.143^***^	−0.091^***^	0.234^***^
Inpatient PHU	−0.220^***^	−0.091^***^	−0.129^*^
Outpatient PHU	−0.247^***^	−0.091^***^	−0.156^***^

Source: authors’ calculation. SEs are robust, clustered and take into consideration SLIHS 2018 survey structure; *P* < 0.1*, *P* < 0.5** and *P* < 0.01***.

**Figure 7.**
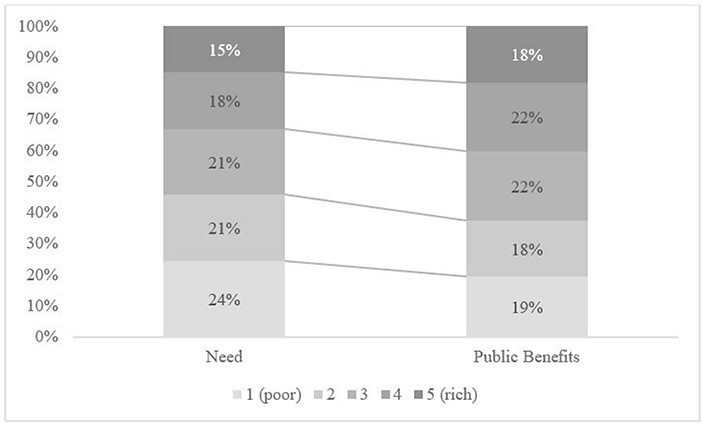
Comparison of needs and benefits

Source: authors’ calculation.

Figure 5 shows that there is a misalignment between the distributions of needs and public healthcare benefits, and this is confirmed by their CIs: the difference between the two CIs is positive and statistically different from zero (+0.099, *P* < 0.01). In other words, total public healthcare benefits are not distributed to the Sierra Leonean population according to their needs (Figure 7). This is driven by two different trends: PHU benefits are pro-poor when compared with needs, and hospital benefits are pro-rich when compared with needs. Hospital outpatient benefits remain pro-rich when compared with needs, while inpatient hospital benefits, which showed a non-significant pro-rich bias versus the line of equality, exhibit a significant pro-rich bias compared with needs (Table 5, benefits needs index 0.128, *P* < 0.01).

In Appendix 2 we extend the benefit incidence analysis including private healthcare providers. When private healthcare providers are included, the overall public and private health benefits distribution are markedly pro-rich.

Finally, the result of the RIF-CI-OLS decomposition (see Appendix 3) shows that an increase in the proportion of households’ residence in rural locations (vs. urban) (association with benefits CI: +0.188, *P* < 0.1, effect on CI of an increase in 1% in proportion of rural households: +5%), and household size, for household sizes between 5 and 7 members (association between increase in proportion of households with size 5 and 6–7 members, and CI of public healthcare benefits: 5 members, +0.31, *P* < 0.01, 6–7 members, +0.35, *P* < 0.01, effect on CI of an increase in 1% in proportion of 5 and 6–7 members households, respectively: +8%, +10%), have the largest influence on the CI of public healthcare benefits.

Although the weighted OLS results show larger marginal effects when compared with the unweighted OLS results, the results are otherwise generally consistent with the unweighted OLS results across all covariates in terms of sign, significance and coefficient magnitudes.

### Redistributive effect of the public healthcare system

**Figure 8.**
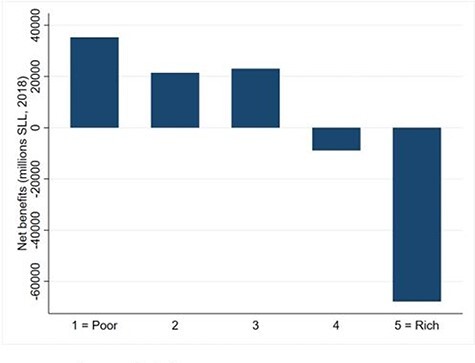
Net public healthcare benefits incidence across income quintiles

Source: authors’ calculation.

Net public healthcare benefits (i.e. public healthcare subsidies minus public healthcare contributions, Figure 8) show that the health system redistributes resources from better off quintiles to worse off quintiles.

Figure 8 shows that the two richest quintiles contribute more to the public health system than what they receive in benefits, making them net contributors. Conversely, the two poorest quintile and the central quintile receive more benefits compared with their contributions, and are net receivers. This finding indicates that the Sierra Leone public healthcare system redistributes resources from the richest quintiles to the poorest ones.

Table 6, row one, shows that the reduction in income inequality induced by public health financing is minimal. This confirms the finding that public health financing is neither progressive nor regressive.

**Table 6.** Redistributive effects of health financing, and public healthcare system, by level

#	Redistributive effect of: ↓	Gini market income| Gini final income	Reduction in Gini index driven by public health system (%)	Percentage of benefits over total benefits
1	Health financing	0.3810 |0.3808	−0.0 percentage points (0.0%)	n.a.
2	Public healthcare system	0.3810 |0.3792	−0.2 percentage points (0.5%)	100%
3	PHU level	0.3810 |0.3798	−0.1 percentage points (0.2%)	46%
4	Secondary/tertiary level	0.3810 |0.3800	−0.1 percentage points (0.2%)	54%

Source: authors’ calculation. The percentage to PHUs is measured using NHA data for 2018: providers of ambulatory and preventive services are considered PHU level, and hospitals are considered the secondary and tertiary level.

Table 6, rows two to four, presents the redistributive effect of the entire public healthcare system (i.e. health financing and benefits provision, all levels considered), further broken down in PHU level and secondary/tertiary level. Both the PHU and secondary/tertiary health system level contribute to redistributing resources and reducing income inequality. In addition, we note that the PHU level delivers a similar reduction in inequality while providing substantially less benefits, than the secondary/tertiary levels.

## Discussion

Achieving UHC is a key target in the Sierra Leone National Health Sector Strategic Plan 2021–2025, which aims to ensure that the entire population of Sierra Leone can access the healthcare they need without suffering financial hardship, regardless of their socioeconomic status. Moreover, reducing income inequalities is also an explicit target of the Sierra Leone Medium Term National Development Plan 2019–2023 (Government of Sierra Leone, 2019). While some evidence supports the idea that investments in health systems drive economic growth (How investing in health has a significant economic payoff for developing economies Remes *et al.*, 2020; Wang, 2015; Piabuo and Tieguhong, 2017; Raghupathi and Raghupathi, 2020; Gaies, 2022), it is less clear whether investments in health systems reduce income inequalities. To what extent the Sierra Leone public healthcare system is equitable, and does it redistribute resources from the rich to the poor? To answer these questions, we analyse the equity, as defined by Ataguba and Akazili (Ataguba and Akazili, 2010), of the Sierra Leone public healthcare system, in both financing and benefit delivery. It is crucial that benefit incidence analysis and financing incidence analysis are conducted together to assess whether a healthcare system is equitable (Ataguba and Akazili, 2010). We then extend these analyses by measuring the redistributive effect induced by the public healthcare system (i.e. fiscal incidence analysis).

Our financing and benefit incidence findings are similar to a recent systematic review of benefit and financing incidence analyses in LMICs (Ataguba *et al.*, 2018), which also found that direct taxes show a progressive distribution, indirect taxes show a regressive distribution, public PHC benefits incidence is usually pro-poor and public hospitals benefits incidence is usually pro-rich. However, in the case of Sierra Leone, benefits provision does not align with needs; therefore, the public health system could be more equitable.

As it was the case for the benefit incidence analysis, the public healthcare system redistributive effect is driven by PHUs, rather than the secondary/tertiary healthcare system level (see Table 6). This is because PHU benefits are pro-poor, while secondary/tertiary benefits are pro-rich. The magnitude of the redistributive effect in Sierra Leone is comparable to that observed in other countries (e.g. Ethiopia [Lustig, 2015], Georgia, Armenia, Indonesia and Jordan [Inchauste and Lustig, 2017b]) and could be enhanced by increasing investments in the public health system, focusing on the PHU level.

In the low-income countries group, it was found that all taxes and subsidies resulted in negative net benefits for the poorest households (World Bank, 2022); therefore, the Sierra Leone public healthcare system is comparatively more favourable to the poorest quintiles than other low-income countries. In the same review (World Bank, 2022), investments in health were listed as ‘high value’ for reducing inequalities: our results confirm this point.

The first policy implication of this study is to prioritize PHU services within the public health sector budget to improve the equity and redistributive effect of the public healthcare system. Conversely, prioritization of hospital services might result in a less equitable public healthcare system. The second policy implication is that increasing the public health sector budget would contribute to the reduction income inequality in Sierra Leone.

The government could also consider policies that increase direct tax revenues and reduce indirect tax revenues to enhance equity, pro-poorness and redistribution induced by public health financing, given that our findings show that direct taxation is more effective than indirect taxation in improving the equity and redistributive effect of the public health system.

The health sector might not be ‘best sector buy’ for the Government of Sierra Leone to reduce income inequality in Sierra Leone. To determine whether the health sector is the most efficient investment to reduce income inequality, we would need to compute the redistributive effect across sectors, which is beyond the scope of this analysis. Expanding this same analysis to other sectors (e.g. education, social protection and non-social sectors) may be of particular interest to policymakers allocating resources across sectors to reduce income inequality in Sierra Leone. Fiscal incidence for public services delivering public goods (e.g. national defence) is also a largely unexplored research area (Lustig, 2019).

An important contribution of this paper is to merge the literature on benefit and financing incidence analysis (McIntyre and Ataguba, 2011; Ataguba *et al.*, 2018) with the fiscal incidence literature on the effect of (public) health systems on income inequality (Lustig, 2015; Inchauste and Lustig, 2017b): to the best of our knowledge, this is the first paper to do that. Moreover, we explore the equity and redistributive effect of the public healthcare system in Sierra Leone, a country for which this knowledge is not available. To the best of our knowledge, this is also the first paper to measure the redistributive effect across health system levels (PHC and secondary/tertiary healthcare): the findings across health system levels might be relevant for other countries advocating for increased PHC financing.

Several limitations should be considered. For this paper, other sectors (e.g. education) are out of scope, and could be considered to compare redistributive effects across sectors. Another limitation is that SLIHS 2018 does not differentiate among different hospitals (e.g. secondary district and regional hospitals, versus tertiary referral hospitals), which might have substantially different unit costs and utilization patterns, nor it provides detail on PHC services provided by hospitals: such detail would have greatly benefited the usefulness of the findings for policy-makers. Measuring healthcare needs in LMICs using self-reported illness consistently under-estimate the needs of lower income households, for various reasons including limited knowledge and the fact that poorer households cannot afford to be sick (Sierra Leone Ministry of Health and Sanitation, 2021). While we included non-injury-related healthcare needs in our healthcare needs measure, it is very likely that healthcare need is more concentrated in poorer households than what we have measured. As noted already, we have computed values for ‘inpatient PHU services’ as households declared being in PHUs overnight, despite PHUs are not supposed to provide inpatient services. Importantly, for utilization and costs data, we used SLIHS 2018 and NHA 2018 and we did not use the government Health and Financial Management Information Systems: using these different data sources could change the results. Finally, as in other benefit incidence analyses, quality of care has not been taken into consideration when the monetary values of benefits were computed (Asante *et al.*, 2020).

Despite these caveats, we believe this research is important for three key reasons. First, it underscores the necessity of sustained investments in PHC to enhance both health equity and income equality. Second, it contributes to the limited literature on financing, benefit and fiscal incidence analyses in Sierra Leone. Lastly, it demonstrates how benefit (McIntyre and Ataguba, 2011), financing (Ataguba *et al.*, 2018) and fiscal incidence (Lustig, 2015; 2019) methods can complement each other, providing policy-relevant insights that can inform decision-making processes.

## Figures and Tables

**Figure A1.**
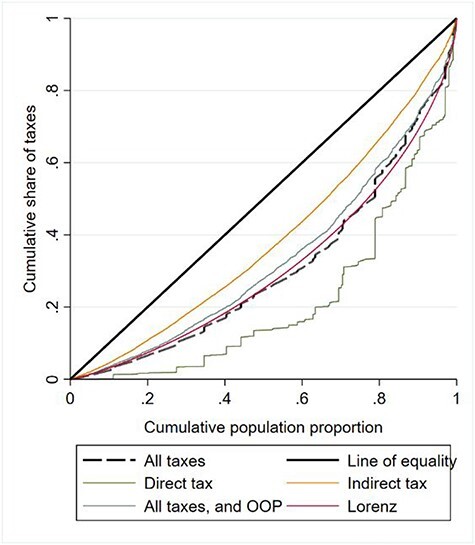
Financing incidence, concentration curves including OOP health expenditures

**Figure A2.**
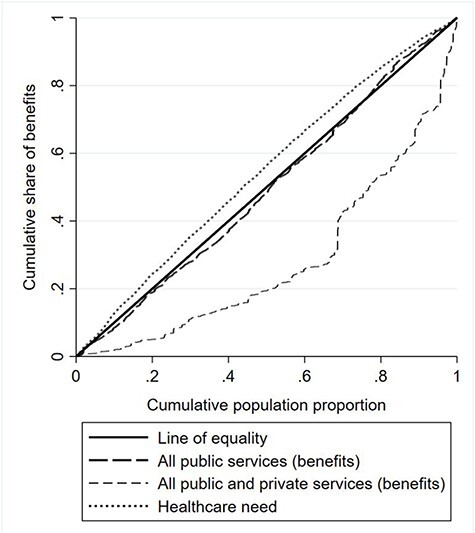
Benefit incidence, concentration curves including private providers

## Footnotes

1. In the fiscal incidence and public finance literature, health services are usually called ‘in kind transfers’ (Inchauste and Lustig, 2017a)

2. CIs have been defined in eq. [1] with reference to public healthcare system financing contributions. For benefits, the measurement is exactly the same, except that $T$ contributions with $B$ benefits, yielding: $CI(B\,|\,Y) = \frac{2}{{\bar B}}cov\left( {{b_i},{R_i}} \right)$

3. In an extreme case where benefits are all concentrated in the poorest (richest) individual, and need is all concentrated in the richest (poorest) individual, then the value of $C{I_{benefits}}$ and would be, respectively, −1 (+1) and +1 (−1), and their difference would be −2 (+2).

## Reflexivity statement

The majority of the co-authors are from a low- and middle-income country (LMIC) and are people of colour. Two co-authors are from Sierra Leone, are based in a Sierra Leone university or government organization and are Sierra Leone economists. The research reflects local priorities: the Ministry of Health and Sanitation has been working on a separate benefit incidence analysis in 2022, and one of the co-authors is the principal health economist at the Ministry of health and Sanitation. The corresponding author is from a high-income country but has substantial professional experience in health financing in LMICs and in Sierra Leone. The research idea originated during the corresponding author PhD period, and during work in Sierra Leone in collaboration with two of the co-authors. The co-authors span different levels of seniority: among the co-authors there is a PhD student, a research fellow and a head of department of economics. Different types of organizations are represented: co-authors work at universities in Sierra Leone and United Kingdom, Ministry of Health and Sanitation and as World Bank consultant.

## Appendix 1. Detailed assumptions on financing and benefit incidence analyses and health system expenditures

Below is an overview of all taxes estimated via SLIHS as per methods section, their weight as % of total taxes and assumptions for taxes not estimated via SLIHS 2018.

**Table A1.** Detailed measurement of cost per units

	NHA 2018, total health expenditure (SLL millions)	% of hospitals and PHU expenditure paid by government	SLL per 1 US$ in 2018 (average)	Total cost (US$)	Utilization units: inpatient nights, outpatient episodes (source: SLIHS 2018)	Cost per unit
Hospital outpatient	787 268.841	5%	7712	5 273 013	2 785 056	1.89
Hospital inpatient	880 233.6184	5%	7712	5 895 677	1 324 468	4.45
PHU outpatient	143 121.9533	14%	7712	2 566 177	7 623 852	0.34
PHU inpatient (average of hospital inpatient and PHU outpatient)						2.39

Source: authors’ calculation.

**Table A2.** Financing incidence detailed assumptions

Tax	Tax base	Tax applied	Explanation
Indirect tax: goods and services tax	All expenditures except rice, books, fuel, transport	15%	15% is the goods and services tax rate for all goods and services in Sierra Leone
Indirect tax: fuel excise duties	Fuel expenditures	9%	Assumption taken from World Bank/Statistics Sierra Leone 2014 report (World Bank, 2014)
Indirect tax: goods and services tax	‘Public transport’ expenditures	30% of total cost assumed to be fuel, then taxed at 9%, therefore 2.7% of total public transport cost	It is assumed that 30% of total public transport ticket is fuel
Direct tax: income tax	Salary for individuals stating that they have a formal contract. Salary is annualized	According to First Schedule of National Income Tax Act (old Leones): Below Le 3 600 001.00 per annum: Nil Le 3 600 001.00 to Le 7 200 000 per annum: 15% Le 7 200 001.00 to Le 10 800 000 per annum: 20% Over 10 800 001.00: 30%	None

**Table A3.** Overview of all assumptions made for financing incidence analysis

	Tax	2018 (% of GDP) (source: Sierra Leone Budget and Finance Act 2019)			
Nomenclature used in paper	Total	14.3	% of total	% of tax Sierra Leone budget sub-group	
Direct tax	**Income taxes**	5.2	36%	100%	
	Of which: Personal	4.1	29%	79%	Estimated using SLIHS^a^
	Of which: Corporate	1	7%	19%	Assumed distributed as personal income tax
Indirect tax	**Goods and services tax (GST)**	2.7	19%	100%	
	Of which: Domestic	1.6	11%	59%	Estimated using SLIHS**
	Of which: Import	1.1	8%	41%	Assumed distributed as indirect tax SLIHS estimate (petroleum and domestic GST). There is no information on SLIHS as to whether goods are imported or not, except for rice.
	**Excise taxes**	3.4	24%	100%	
	Of which: Petroleum products	1.5	10%	44%	Estimated using SLIHS**
	Of which: Import duties	1.8	13%	53%	Assumed distributed as indirect tax SLIHS estimate (petroleum and domestic GST). There is no information on SLIHS as to whether goods are imported or not, except for rice.
Other	**Mines department**	0.7	5%	21%	Assumed to be distributed as all other revenues
	**Other departments**	2.3	16%	68%	Assumed to be distributed as all other revenues

a Estimated value via SLISH 2018 was 83% of the total amount stated in the Sierra Leone 2019 Budget Act; therefore, it was adjusted to reflect the actual Budget Act value. We note that six households with reported income from approximately 250 000 USD to 650 000 USD have been removed as they are likely reporting mistakes: their jobs are paid monthly (e.g. government job), but they reported being paid hourly instead of monthly, resulting in over-estimation.

** Total indirect taxes estimated via SLIHS were 87% of total indirect taxes as per Sierra Leone 2019 Budget Act; therefore, it was adjusted to reflect the actual Budget Act value.

**Table A4.** Detail of public health expenditures across health providers

Financing schemes: HF.1				
Health providers	Government schemes and compulsory contributory health care financing schemes			As% of total
**HP.1**		**Hospitals**	86 760.0	39%
	HP.1.1	General hospitals	25 586.3	12%
	HP.1.2	Mental health hospitals	1734.8	1%
	HP.1.3	Specialized hospitals (Other than mental health hospitals)	2905.0	1%
	HP.1.nec	Unspecified hospitals (n.e.c.)	56 533.9	26%
**HP.3**		**Providers of ambulatory health care**	29 463.0	13%
	HP.3.4	Ambulatory health care centres	29 463.0	13%
**HP.5**		**Retailers and Other providers of medical goods**	509.0	0%
	HP.5.1	Pharmacies	509.0	0%
**HP.6**		**Providers of preventive care**	44 277.3	20%
**HP.7**		**Providers of health care system administration and financing**	52 215.5	24%
	HP.7.1	Government health administration agencies	52 215.5	24%
**HP.9**		**Rest of the world**	7819.3	4%
**All HP**			221 044.2	100%

## Appendix 2. Robustness checks and extensions of the benefit and financing incidence analyses

**Table A5.** Computed values of benefits from WHO CHOICE and NHA 2018

WHO CHOICE unit cost definition and computation	SLIHS 2018 definition	Computed value (US$) from WHO CHOICE	Computed value (US$) from NHA 2018
Average of health centre outpatient with bed, and without bed	PHUs outpatient	2.325	0.34
Average of primary hospital inpatient and outpatient health centre with bed	PHUs inpatient	5.88	2.39
Average of secondary and tertiary hospital outpatient	Hospitals outpatient	3.13	1.89
Average of secondary and tertiary hospital inpatient	Hospitals inpatient	10.98	4.45

Source: authors’ calculations.

### Use WHO CHOICE for benefits

We re-do the benefit incidence analysis using WHO CHOICE 2021 data instead of NHA 2018 as the source for outpatient and inpatient visits values in US$. As shown in the table below, the NHA values are different from WHO CHOICE values. For this reason, using WHO CHOICE result in overall benefits being slightly pro-poor and the public healthcare system of Sierra Leone being more equitable. NHA 2018 data, which are collected from development partners, governments and from household surveys, are to be preferred from WHO CHOICE 2021, which is modelled from NHA across countries.

**Table A6.** Concentration index using different benefits costs, from WHO CHOICE

Public benefits	Concentration index (CI)	CI—WHO CHOICE
All public benefits	0.008	−0.079^***^
Inpatient hospital	0.037	0.036
Outpatient hospital	0.143^***^	0.116^***^
Inpatient PHU	−0.220^***^	−0.199^***^
Outpatient PHU	−0.247^***^	−0.245^***^

Source: authors’ calculations. SEs are robust, clustered and take into consideration SLIHS 2018 survey structure; **P* < 0.1, ***P* < 0.05, ****P* < 0.01.

### Financing and benefit incidence analysis: include OOP health expenditures and private healthcare providers

Kakwani index of all taxes and OOP health expenditures: −0.047, *P* < 0.01 => regressive health financing incidence, when including OOP health expenditures

**Table:**

	Kakwani index	SE	Weights
**Government public healthcare expenditure**	0.011	0.006	13%
**OOP health expenditures**	−0.055	0.002	87%
**Weighted average**	−0.046	0.005	

Notes: the NHA 2018 do not sum up to 100% because the remaining part (35%) is external expenditures, which have been ignored in this analysis, as done in the literature.

Concentration index of all benefits including from private healthcare providers: 0.44, *P* < 0.01, substantially pro-rich

### Using additional OOP costs

User fees collected by health service providers at the PHU level and, to some extent, at the secondary/tertiary level, are largely informal and expected to fund volunteer health workers (Witter *et al.*, 2016) (i.e. health workers without a government salary). In our main results, we subtract from public benefits consultations costs, and costs to stay in the hospital (for inpatient services) paid OOP by patients to providers as user fees. In this robustness check, in addition to consultations and costs to stay in the hospital, we subtract from public benefits also drugs and tests costs paid OOP by patients to the providers as user fees. The CIs are largely unchanged.

**Table A7.** Concentration index with different definition of user fees

	Concentration index	CI—increased
Public benefits	(CI)	user fees
All public benefits	0.008	−0.01
Inpatient hospital	0.037	0.030
Outpatient hospital	0.143^***^	0.102^***^
Inpatient PHU	−0.220^***^	−0.225^***^
Outpatient PHU	−0.247^***^	−0.311^***^

Source: authors’ calculations. SEs are robust, clustered and take into consideration SLIHS 2018 survey structure; **P* < 0.1, ***P* < 0.05, ****P* < 0.01.

### Absolute CIs

Standard CI is a measure of relative inequalities. If benefits are increased by the same percentage to all households, there will be no difference in the standard CI. However, there would be a difference in absolute inequality, which we can measure via the generalized CI (also called absolute CI), computed as the standard CI times the average of the benefits variable. Generalized CIs in SLL are provided in the table below.

**Table A8.** Absolute CIs

Public benefits	Standard CI (CI)	Generalized CI (SLL)
All public benefits	0.008	2144
Inpatient hospital	0.037	1491
Outpatient hospital	0.143^***^	4697
Inpatient PHU	−0.220^***^	−896
Outpatient PHU	−0.247^***^	−2891

Source: authors’ calculations. SEs are robust, clustered and take into consideration SLIHS 2018 survey structure; **P* < 0.1, ***P* < 0.05, ****P* < 0.01.

## Appendix 3. Recentred influence function and OLS, detailed methodology and results

Methods described in this section are largely building from Heckley *et al.* 2016 (Heckley *et al.*, 2016).

We first define a bivariate index $I$ as:

(8)
$$I = {v^I}\left( {{F_{B,{R_i}}}} \right) = {v^{wI}}\left( {{F_B}} \right){v^{AC}}\left( {{F_{B,{R_i}}}} \right)$$

where $B$ are public healthcare benefits, ${R_i}$ is the fractional rank of each household based on expenditure per adult equivalent $Y$ (therefore, ${R_i}$ is equivalent to ${F_Y}$ the CDF of $Y$), ${v^{wI}}\left( {{F_B}} \right)$ is a weighting function required to measure a particular version of the CI (standard, Wagstaff or Erreygers) and ${v^{AC}}\left( {{F_{B,{F_{SES}}}}} \right)$ is the absolute CI AC:

(9)
$${v^{AC}}\left( {{F_{B,{R_i}}}} \right) = 2cov\left( {B,\,{R_i}} \right)$$

The standard, Wagstaff and Erreygers CIs have the same AC but different weights. For the standard CI ${v^{wI}}\left( {{F_B}} \right) = 1/\bar B$. Substituting ${v^{wI}}\left( {{F_B}} \right) = 1/\bar B$ and ${v^{AC}}\left( {{F_{B,{R_i}}}} \right) = 2cov\left( {B,{R_i}} \right)$ to eq. (8) yields the CI defined in section 2.3, footnote 1, defined as $CI = \frac{2}{{\bar B}}cov\left( {B,{R_i}} \right)$. Because healthcare benefits are not dummy and are not bounded upwards, we prefer the standard CI to the Erreygers and Wagstaff CI which, defining benefits upper bound and lower bounds as ${B_{ub}}$ and ${B_{lb}}$, respectively, can be measured by changing the CI weight. For the Erreygers CI, ${v^{wI}}\left( {{F_B}} \right) = 4/\left( {{B_{ub}} - {B_{lb}}} \right)$, and for the Wagstaff CI ${v^{wI}}\left( {{F_B}} \right) = \frac{{{B_{ub}} - {B_{lb}}}}{{({B_{ub}} - \bar B)\left( {\bar B - {B_{lb}}} \right)}}$ .

Now that we have defined CIs, we can define influence functions (IF). Let ${G_{b,{F_Y}}}$ be a distribution function (bivariate) obtained by an infinitesimal contamination of ${F_{B,{F_Y}\left( y \right)}}$ in both $b\,$and ${F_Y}\left( y \right)$:

(10)
$${G_{b,{F_Y}\left( y \right)}} = \left( {1 - \varepsilon } \right){F_{B,{F_Y}}} + \varepsilon {\delta _{b,{F_Y}\left( y \right)}}$$

${G_{b,{F_Y}}}$ is in fact a distribution that is $\varepsilon $ away from the original distribution ${F_{B,{F_Y}}}$ in the direction of ${\delta _{b,{F_Y}\left( y \right)}}$. $\varepsilon $ is a weight, or probability, representing the relative change driven by the addition of ${\delta _{b,{F_Y}\left( y \right)}}$, which is defined as:

(11)
$${\delta _{b,{F_Y}\left( y \right)}}\left( {l,r} \right) = \left\{ {\begin{array}{*{20}{c}}
{0\ if\ l \lt b\ or\ r \lt {F_Y}\left( y \right)}\\
{1\ if\ l \ge b\ and\ r \ge {F_Y}\left( y \right)}
\end{array}} \right.$$

where $l$ is a draw from $B$ and $r$ is a draw from ${F_Y}$.

We can now define the bivariate influence function (IF) at point $b,\,{F_Y}\left( y \right)$ as:

(12)
$$IF\left( {b,\,{F_Y}\left( y \right);\,{v^I}} \right) = \mathop {\lim }\limits_{\varepsilon \to 0} \frac{{{v^I}\left( {{G_{b,{F_Y}\left( y \right)}}} \right) - {v^I}\left( {{F_{B,{F_Y}}}} \right)}}{\varepsilon }$$

The recentred influence function (RIF) can simply be thought of as a minor extension of the IF, obtained by summing the original function to the IF, thus ‘recentring’ it towards the original function.

(13)
$$RIF\left( {b,\,{F_Y}\left( y \right);\,{v^I}} \right) = {v^I}\left( {{F_{B,{F_Y}}}} \right) + IF\left( {b,\,{F_Y}\left( y \right);\,{v^I}} \right)$$

That is, the contribution of observation $b,\,{F_Y}\left( y \right)$ to the distribution of ${v^I}$, which in our case is the standard CI of public healthcare benefits ranked by household expenditure per adult equivalent.

Following Hackley *et al.* 2016, and because we defined ${v^I}\left( {{F_{B,{F_Y}}}} \right) = {v^{wI}}\left( {{F_B}} \right){v^{AC}}\left( {{F_{B,{F_Y}}}} \right)$, the RIF of CI should take into consideration both index weights and absolute concentration, and is:

(14)
$$RIF\left( {b,\,{F_Y}\left( y \right);\,{v^{CI}}} \right) = \overbrace {{v^{CI}}\left( {{F_{B,{F_Y}}}} \right)}^{CI} + \overbrace {\frac{{\left( {\bar B - b} \right)}}{{{{\bar B}^2}}}}^{\begin{array}{*{20}{c}}
{IF\,of\,weight\,}\nonumber\\
{function}
\end{array}}\nonumber\\{v^{AC}}\left( {{F_{B,{F_Y}}}} \right) + \left( {\frac{1}{{\bar B}}} \right)IF\left( {b,\,{F_Y}\left( y \right);\,{v^{AC}}} \right)$$

where $IF\left( {b,\,{F_Y}\left( y \right);\,{v^{AC}}} \right) = - 2{v^{AC}}\left( {{F_{B,{F_Y}}}} \right) + \,\bar B - b - 2b{F_Y}\left( y \right)$$- 2\smallint \limits^{y} \smallint \limits^{+ \infty} b{F_{B,{F_Y}}}dbd{F_Y}\left( y \right)$

For the proofs of the above equations, we refer the reader to the appendix of Hackley *et al.* 2016.

### RIF regression decomposition

Following Firpo *et al.* 2009 (Firpo *et al.*, 2009), and assuming linearity in the relationship between the RIF and covariates, we can use OLS to complete a RIF regression decomposition. RIF values are used as the dependent variable; therefore,

(15)
$$RIF\left( {b,\,{F_Y}\left( y \right);\,{v^I}} \right) = {\bf{\mathit{X}}}\beta + {\varepsilon _i}\,\,\,\,\,\,\,\,\,\,\,E\left[ {{\varepsilon _i}} \right] = 0\,\,\,$$

where ${\bf{\mathit{X}}}$ is a vector of covariates. The recentring of the RIF means that ${v^I}\left( {{F_{B,{F_Y}}}} \right) = E\left[ {RIF\left( {b,\,{F_Y}\left( y \right);\,{v^I}} \right)} \right]$, and therefore:

(16)
$$E[RIF\left( {b,\,{F_Y}\left( y \right);\,{v^I}} \right)\left] { = E} \right[{\bf{\mathit{X}}}\beta \left] { + E[{\varepsilon _i}} \right] = \overline {\bf{\mathit{X}}} \beta $$

The unconditional partial effect $\beta $ on the CI is then:

(17)
$${\beta _k} = \frac{{d{v^I}\left( {{F_{B,{F_Y}}}} \right)}}{{d{{\overline {\bf{\mathit{X}}} }_{\bf{\mathit{k}}}}}}$$

This can be interpreted, for continuous variables, as the effect ${\beta _k}\,$of an increase in one unit in the unconditional expectation ${\overline {\bf{\mathit{X}}} _{\bf{\mathit{k}}}}$ on the CI (${v^I}\left( {{F_{B,{F_Y}}}} \right)$) of public healthcare benefits $B$, measured using expenditure per adult equivalent $Y$ as the living standard measure. For dummy variables (for example, household residing in rural area equal one, zero otherwise), the change from 0 to 1 implied by the OLS regression is equivalent to moving from 0% to 100% of households in rural area; therefore, the coefficient needs to be interpreted carefully. For a binary variable, the CI percentage contribution of an increase of 1 percentage point in the proportion of households belonging to a particular group (e.g. household residing in a rural area) is calculated as:

(18)
$$\frac{{{\beta _k}}}{{CI}}*1\% $$

We remind that $CI = {v^{wI}}\left( {{F_B}} \right){v^{AC}}\left( {{F_{B,{R_i}}}} \right)$ with weight $\frac{1}{{\bar B}}$.

The benefits of using the RIF-CI-OLS methodology (Heckley *et al.*, 2016) versus the ‘standard’ CI decomposition methodology from Wagstaff, Van Doorslaer, Watanabe (Wagstaff *et al.*, 2003) are three. First, OLS is a rather familiar methodology and the interpretation of RIF-CI-OLS results is analogous to standard OLS regressions. Second, standard CI decomposition requires more stringent assumptions for identification. More specifically, standard CI decomposition requires that the determinants of health do not determine the rank variable and do not determine the weighting function. Both these assumptions do not appear to be reasonable in our case, as determinants of public health benefits provision (e.g. rural residence, education, employment) are almost certainly determinants of the rank variable (i.e. total household expenditure per adult equivalent), and of the weighting function (i.e. the inverse of average income). While we do not attempt to find causal relationship, we attempt to find associations and therefore the assumptions required are important. The RIF-CI-OLS, for identification of partial unconditional effects require that the CI is differentiable, that the RIF-CI is linear and that the OLS regression errors have mean zero. Finally, standard CI decomposition results are weighting function agnostic: regardless of the CI weighting function, standard CI decomposition would provide the same results. For all these reasons, we believe that RIF-CI-OLS is the preferred methodology for CI decomposition.

To implement the two-step procedure described above, we use the software Stata 17 and the commands egen rifvar and regress (Rios-Avila, 2020).

### Results

**Table A9.** RIF-CI-OLS results

		(Cotlear and Rosemberg, 2018)	(Cotlear *et al.*, 2015)	(World Health Organization, 2019)	(Brundtland, 2022)
	Covariates	OLS	OLS ME	WOLS	WOLS ME
*Residence*	Residence (rural = 1, urban = 0)	0.188^*^	5%	0.239^**^	30%
		(0.106)		(0.120)	
*Income quintile*	Low income quintile	Reference			
	Mid-low income quintile	0.0589	2%	0.0705	9%
		(0.0847)		(0.104)	
	Mid income quintile	0.00747	0%	0.0400	5%
		(0.0863)		(0.101)	
	Mid-high income quintile	0.134	4%	0.136	17%
		(0.107)		(0.121)	
	High income quintile	0.0547	1%	0.0574	7%
		(0.170)		(0.176)	
*HHH age*	HHH age (quartile 1): <36	Reference			
	HHH age (quartile 2): 36−44	0.0432	1%	0.00665	1%
		(0.0443)		(0.0533)	
	HHH age (quartile 3): 45−55	0.0175	0%	−0.00293	0%
		(0.0515)		(0.0554)	
	HHH age (quartile 4): 56 or older	0.0800^*^	2%	0.0536	7%
		(0.0409)		(0.0425)	
*HHH education*	HHH education: none	Reference			
	HHH education: primary	−0.110	−3%	−0.110	−14%
		(0.0928)		(0.103)	
	HHH education: secondary or more	−0.0171	0%	0.000424	0%
		(0.0454)		(0.0459)	
*HHH employment*	HHH: unemployed	Reference			
	HHH: employed, agriculture	−0.0619	−2%	−0.0964	−12%
		(0.105)		(0.122)	
	HHH: employed, all other	−0.0697	−2%	−0.0493	−6%
		(0.0749)		(0.0907)	
*HHH gender*	HHH gender (female = 1, male = 0)	0.0189	1%	0.0365	5%
		(0.0514)		(0.0498)	
*HH size*	HH size (quartile 1): <5	Reference			
	HH size (quartile 2): 5	0.314^***^	8%	0.289^***^	36%
		(0.0542)		(0.0496)	
	HH size (quartile 3): 6−7	0.353^***^	10%	0.340^***^	43%
		(0.0668)		(0.0697)	
	HH size (quartile 4): 8 or more	0.103	3%	0.0553	7%
		(0.110)		(0.104)	
	Constant	−0.232		−0.272^*^	
		(0.146)		(0.141)	
	RIF mean (CI):	0.037		0.008	
	Observations	6810		1 407 531	
	R-squared	0.014		0.015	

Source: authors’ elaboration, data sources described in the methods section and methodology in Appendix 3. Notes: the dependent variable is the RIF of the CI for all public healthcare system benefits. Robust, clustered SEs are used. SEs are bootstrapped using 500 replications. ME stands for marginal effects and are the percentage increase/decrease of the CI driven by an increase in 1% in the relative population sub-group (i.e. rural residents sub-group, mid-low income quintile sub-group, etc.); they are measured as $\frac{\beta }{{CI}}*1\% $. The full estimation process is bootstrapped to calculate SEs. **P* < 0.1, ***P* < 0.05, ****P* < 0.01.

We note that the difference between the RIF mean (i.e. the CI) shown in Table A9, column 1 and the CI shown in Table 4 is driven by SLIHS 2018 weights: this is confirmed by the fact that the CI in Table A9, column 3 (WOLS) is identical to the CI shown in Table 4.
